# SMURF Reconstructs Single-Cells from Visium HD Data to Reveal Zonation of Transcriptional Programs in the Intestine

**DOI:** 10.1101/2025.05.28.656357

**Published:** 2025-07-15

**Authors:** Juanru Guo, Simona Sarafinovska, Ryan A. Hagenson, Mark C. Valentine, Joseph D. Dougherty, Robi D. Mitra, Brian D. Muegge

## Abstract

High-resolution spatial transcriptomics demands new computational tools to accurately assign transcripts to individual cells. We developed SMURF (Segmentation and Manifold UnRolling Framework), a novel soft-segmentation algorithm that maps mRNAs from barcoded capture spots to nearby nuclei. SMURF also “unrolls” cells in complex tissue structures, placing them on standard Cartesian coordinates, thus empowering studies of cellular organization in tissues. Using SMURF, we identified the zonation of gene expression programs along the maturing intestinal villus and the transcription factors that regulate them. We investigated how functionally important proximal-to-distal gene expression gradients in the intestine are specified. We discovered that gene expression gradients along the proximal-distal axis accumulate in the villus tip and that tip gene expression is reprogrammed by environmental signals in the lumen, suggesting environmental inputs are a major determinant of regional transcriptional identity. These results demonstrate SMURF provides a powerful toolbox for analysis of regional transcriptional programs along tissue manifolds.

## Full Text Availability

The license terms selected by the author(s) for this preprint version do not permit archiving in PMC. The full text is available from the preprint server.

